# The role of nodes in arsenic storage and distribution in rice

**DOI:** 10.1093/jxb/erv164

**Published:** 2015-04-28

**Authors:** Yi Chen, Katie L. Moore, Anthony J. Miller, Steve P. McGrath, Jian Feng Ma, Fang-Jie Zhao

**Affiliations:** ^1^Department of Sustainable Soils and Grassland Systems, Rothamsted Research, Harpenden, Hertfordshire AL5 2JQ, UK; ^2^School of Materials, University of Manchester, Manchester M13 9PL, UK; ^3^Department of Metabolic Biology, John Innes Centre, Norwich Research Park, Norwich NR4 7UH, UK; ^4^Institute of Plant Science and Resources, Okayama University, Chuo 2-20-1, Kurashiki 710-0046, Japan; ^5^State Key Laboratory of Crop Genetics and Germplasm Enhancement, College of Resources and Environmental Sciences, Nanjing Agricultural University, Nanjing, 210095, China

**Keywords:** Arsenic, dimethylarsinic acid, Lsi2, node, rice, synchrotron μX-ray fluorescence.

## Abstract

Knowledge of arsenic (As) accumulation in rice (*Oryza sativa* L.) is important for minimizing As transfer to the food chain. The aim of this study was to investigate the role of rice nodes in As storage and distribution. Synchrotron μX-ray fluorescence (μ-XRF) was used to map As distribution in the top node and internode of a *lsi2* mutant defective in silicon/arsenite efflux carrier and its wild-type (WT) grown in soil. *Lsi2* expression in different tissues during grain filling was investigated by quantitative RT-PCR. Arsenite or dimethylarsinic acid (DMA) was supplied to excised panicles to investigate the roles of Lsi2 and phytochelatins (PC) in As distribution. μ-XRF mapping revealed As storage in the phloem of different vascular bundles in the top node and internode. Soil-grown plants of *lsi2* had markedly decreased As accumulation in the phloem compared with the WT. *Lsi2* was strongly expressed, not only in the roots but also in the nodes. When excised panicles were exposed to As(III), the *lsi2* mutant distributed more As to the node and flag leaf but less As to the grain compared with the WT, while there was no significant difference in DMA distribution. Inhibition of PC synthesis by l-buthionine-sulphoximine decreased As(III) deposition in the top node but increased As accumulation in the grain and flag leaf. The results suggest that rice nodes serve as a filter restricting As(III) distribution to the grain. Furthermore, Lsi2 plays a role in As(III) distribution in rice nodes and phytochelatins are important compounds for As(III) storage in the nodes.

## Highlight

Rice nodes serve as an important filter restricting arsenite distribution to the grain.

## Introduction

Rice is the staple food for about half of the world’s population, but it is also the most important dietary source of inorganic arsenic (As), a class-one carcinogen (Meharg *et al.*, 2009; Li *et al.*, 2011). Paddy rice accumulates As more efficiently than other cereals (Williams *et al.*, 2007; Su *et al.*, 2010) because the bioavailability of As in paddy soil is elevated due to the mobilization of arsenite (As(III)) under the anaerobic conditions (Xu *et al.*, 2008; Stroud *et al.*, 2011) and the uptake of As(III) via the silicic acid transporters which are strongly expressed in rice roots (Ma *et al.*, 2008). Paddy water management, and selection and breeding of low-As-accumulating cultivars are two of the possible measures that may be used to decrease As accumulation in rice (Zhao *et al.*, 2010). A better mechanistic understanding of As uptake and translocation would enable the development of strategies to minimize the transfer of As to the food chain.

The mechanism of As uptake and transport in plants depends on the chemical species of As. Arsenite, the dominant species of As in anaerobic soils, is taken up mainly through two silicic acid transporters, Lsi1 and Lsi2, in rice roots (Ma *et al.*, 2008). Lsi1 is an aquaporin channel belonging to the nodulin26-like intrinsic protein (NIP) family. It is localized on the plasma membranes of the distal side of both exodermal and endodermal cells, and allows silicic acid and As(III) to permeate into the cells (Ma *et al.*, 2006). Lsi2 is an efflux transporter localized in the plasma membranes of the proximal side of the exodermal and endodermal cells (Ma *et al.*, 2007), mediating the efflux of silicic acid and As(III) towards the stele for xylem loading (Ma *et al.*, 2008). Mutation of Lsi2 has a greater impact than Lsi1 mutation on As accumulation in the above-ground tissues of rice grown under field conditions. Silicic acid also competes with As(III) uptake by rice (Ma *et al.*, 2008). Owing to this competition and a down-regulation of *Lsi1* and *Lsi2* expression by Si supply, Si fertilizers have been shown to suppress the accumulation of inorganic As by rice plants (Li *et al.*, 2009b; Seyfferth and Fendorf, 2012; Liu *et al.*, 2014). Lsi1 is also partly responsible for the uptake of undissociated monomethylarsonic acid (MMA) and dimethylarsinic acid (DMA) (Li *et al.*, 2009a), which are produced by soil microorganisms and may accumulate in rice grain (Lomax *et al.*, 2012). By contrast, arsenate [As(V)] is taken up by rice roots via phosphate transporters (Wu *et al.*, 2011). As(V) typically accounts for between 5% and 20% of the total As in the soil solution under flooded conditions (Khan *et al.*, 2010), but becomes the predominant species when water is drained (Xu *et al.*, 2008; Li *et al.*, 2009b). Once taken up inside plant cells, As(V) is readily reduced to As(III) (Zhao *et al.*, 2009). It was previously thought that the plant protein ACR2 (also called CDC25), a homologue of the yeast arsenate reductase ACR2, was responsible for As(V) reduction (Dhankher *et al.*, 2006; Duan *et al.*, 2007). Recent studies have shown that ACR2 plays no role in As metabolism in *Arabidopsis thaliana* (Liu *et al.*, 2012; Chao *et al.*, 2014). A new arsenate reductase, named HAC1 (Chao *et al.*, 2014) or ATQ1 (Sanchez-Bermejo *et al.*, 2014), has recently been identified in *A*. *thaliana*. This enzyme is critical for limiting As accumulation in *A*. *thaliana* shoots; the loss of function of HAC1 results in decreased As(V) reduction in the roots, diminished As(III) efflux to the external medium and hyperaccumulation of As in the shoots (Chao *et al.*, 2014).

Inorganic As has a relatively low mobility in non-hyperaccumulator plants. For example, a short-term (2–4 d) experiment using ^73^As as a tracer showed that only 10% of the As(III) taken up by rice roots was distributed to the shoots, and 3.3% of the ^73^As in the shoots was distributed to the grain (Zhao *et al.*, 2012). This low mobility is attributed to the formation of As(III)–phytochelatin complexes and their subsequent sequestration in the vacuoles (Raab *et al.*, 2007; Liu *et al.*, 2010; Moore *et al.*, 2014). Using synchrotron micro-focused X-ray fluorescence (μ-XRF) and high-resolution secondary ion mass spectrometry (NanoSIMS), Moore *et al*. (2011, 2014) showed a strong co-localization of As and S in the vacuoles of the pericycle and endodermal cells in the roots, and the phloem companion cells in the stem and leaf veins.

Nodes in graminaceous plants are junctional regions where leaf sheaths and branches join the stem. Nodes act as a hub for controlling the preferential distribution of nutrients towards the developing tissues that have high requirements for nutrients (Yamaji and Ma, 2014). Each node is connected to an upper node (or the panicle in the case of node I) and lower nodes through complex and well-organized vascular systems. Three different types of vascular bundles (VBs), namely enlarged VB (EVB), transit VB (TVB), and diffuse VB (DVB), are localized in each node, which facilitates the exchange of nutrients among these vasculatures. Many membrane transporters are highly expressed in nodes and play important roles in the distribution of different elements (Yamaji and Ma, 2014). In the case of As, it has been shown that rice nodes contain a much higher concentration of As than internodes and leaves, probably due to the presence of many vascular bundles in the nodes (Moore *et al.*, 2014). Tonoplast transporters for As(III)–phytochelatin complexes have been identified in both *A*. *thaliana* and rice (Song *et al*., 2010, 2014). In rice, OsABCC1 is localized to the tonoplast in the phloem cells in the node and is responsible for the sequestration of As(III)–phytochelatin in the vacuoles (Song *et al.*, 2014). Thus, nodes appear to be crucial places for As storage and for controlling As distribution to the rice grain (Yamaji and Ma, 2014; Zhao *et al.*, 2014). Compared with As(III), DMA has a greater mobility within plants (Carey *et al.*, 2010, 2011; Ye *et al.*, 2010; Lomax *et al.*, 2012), but the reasons remain unclear.

The objective of the present study was to investigate the role of rice nodes in As storage and distribution to the grain. Synchrotron μ-XRF mapping was used to image the cellular distribution of As in the top node and internode of an Lsi2 mutant (*lsi2*) and the wild type (WT). Short-term feeding experiments were carried out using excised panicles to investigate the effect of Lsi2 mutation and an inhibitor of the synthesis of thiol compounds on the distribution of As(III) and DMA. As(III) and DMA were chosen because they are the most important As species in the rice grain and have contrasting mobility within rice plants.

## Materials and methods

### Plant materials and culture

Plant materials used in the present study included the *lsi2-1* mutant (Ma *et al.*, 2007) and its WT Taichung-65 (T-65), and an early flowering cultivar Italica Carolina, both belonging to the *Japonica* subspecies of rice (*Oryza sativa* L.). Rice seeds were surface-sterilized with 0.5% active NaClO for 15min, rinsed and soaked in deionized water thoroughly, and then placed on a nylon net floating on a 0.5mM CaCl_2_ solution. After germination, seedlings were transferred to either nutrient solution or soil for further growth. For the synchrotron μ-XRF mapping experiment, *lsi2-1* and WT plants were grown in three pots each containing 2kg soil amended with 5mg As kg^–1^ of arsenate. The soil was collected from the plough layer of an arable field on the Rothamsted farm and contained 1.42% organic C, 0.13% total N, and 11.6mg kg^–1^ total As and had a pH of 5.2 (Li *et al.*, 2009*b*). Soil was flooded with deionized water during the plant growth period; under this condition the predominant form of As in the soil pore water was found to be As(III) (Li *et al.*, 2009*b*). Basal nutrients (N, P, and K) were added to the soil as described previously by Li *et al.* (2009b). Plants were grown to the grain-filling stage inside a controlled-environment glasshouse. The climatic conditions were a 12h photoperiod with natural sunlight supplemented with sodium vapour lamps to maintain a minimum light intensity of 350 μmol m^–2^ s^–1^, 25/20 °C day/night temperatures, and 70% relative humidity.

For the experiments investigating As translocation in excised panicles, 10–12 plants were grown in 1.0 l full-strength Kimura nutrient solution (one plant per pot) up to the grain-filling stage. The nutrient composition was as follows: 0.18mM KNO_3_, 0.37mM Ca(NO_3_)_2_, 0.55mM MgSO_4_, 0.18mM KH_2_PO_4_, 0.37mM (NH_4_)_2_SO4, 0.5 μM MnCl_2_, 3 μM H_3_BO_3_, 0.1 μM (NH_4_)_6_Mo_7_O_24_, 0.4 μM ZnSO_4_, 0.2 μM CuSO_4_, 40 μM NaFe(III)-EDTA, and 2mM MES (pH adjusted to 5.5 with NaOH). Nutrient solution was renewed every 5 d. Rice plants were grown in a controlled-environment glasshouse as described above.

### Synchrotron μ-XRF mapping

At the grain-filling stage, node I (top node) and internode I (above the top node, also called the peduncle) of *lsi2-1* and the WT from three individual plants were cut and placed in MES (2-(*N*-morpholino)ethanesulphonic acid) buffer (20mM MES, 2mM CaCl_2_, 2mM KCl, 0.2M sucrose, pH 5.5). These were cut with a razor blade into 0.4–0.5mm thick sections, and placed into a planchette coated with hexadecane. Another planchette was placed on top. Sections were frozen using a Leica HPM100 high-pressure freezer (Leica, Wetzlar, Germany) with a pressure of 210MPa at –196 °C for 30 s. The frozen samples were freeze-substituted, embedded in resin and sectioned to 7 μm thickness as previously described by Moore *et al.* (2014). An adjacent section, 1 μm in thickness, was stained with Toluidine blue for light microscopy inspection of the cellular structure. Synchrotron μ-XRF was undertaken at the Diamond Light Source, UK, on the I18 microfocus beamline. The incident X-ray energy was set to 12.4 keV using a Si(111) monochromator. The X-ray fluorescence spectra of As and other trace elements were collected using a Si drift detector. The beam size and step size were both 5 μm for node I specimens and 2 μm for internode I specimens. Quantification of the concentrations of arsenic and other elements of interest in the samples were carried out using an external calibration with XRF reference materials.

### Quantitative real time RT-PCR (Q-PCR)

Total RNA was extracted from different plant tissues (three biological replicates) of the cv. T-65 at the grain-filling stage grown in hydroponic culture without As supply with an RNeasy Plant Mini Kit (Giagen) following the manufacturer’s instructions. cDNA was synthesized with SuperScript® III Reverse Transcriptase (Invitrogen) and Q-PCR was performed on a ABI Prism 7500 (Applied Biosystems) with SYBR® Green JumpStart™ Taq ReadyMix™ (Sigma). Rice *Actin* and *Histon H3* were used as reference genes. The primers used are as follows: *Lsi2* forward 5′-ATCACCTTCCCCAAGTTCC-3′ and reverse 5′-CAGCTCCCTCCAGTACATGC-3′; *Actin* forward 5′-ATCATGAAGTGCGACGTGGA-3′ and reverse 5′-AATGCCAGGGAACATAGTGGT-3′; *Histon H3* forward 5′-TTGATTCCCCTCTCGCTTCC-3′ and reverse 5′-TGAGTCTTTAACCGAACCCTGT-3′. Relative expression was calculated as previously described by Rieu and Powers (2009). The final expression level in different tissues was standardized by the expression in node I.

### Arsenic translocation in excised panicles

At the grain-filling stage, panicles of *lsi2-1* and its WT (cv. T-65) grown in hydroponic culture were excised at approximately 10cm below node I as previously described (Carey *et al.*, 2010). For each As treatment, six excised panicles of similar size were transferred to plastic bottles (one panicle per bottle) containing 90ml of the full-strength Kimura nutrient solution with 10 μM As(III) or 5 μM DMA. The solution also contained 10 μM RbCl and 10 μM SrCl_2_ as the tracer for phloem and xylem transport, respectively (Carey *et al.*, 2010). Approximately 3cm of the bottom part of the stem below node I was submerged in the solution and the panicles were supported with a sponge at the bottle opening. The bottles were placed inside the same controlled-environment glasshouse used in the pre-culture of rice plants. After 2 d, the stem section below node I was discarded and the rest was separated into node, internode, leaves, husk, and grain for the analysis of As, Rb, and Sr.

In a separate experiment, the effect of l-buthionine-sulphoximine (BSO), a γ-glutamylcysteine synthetase inhibitor, on As translocation in excised panicles was investigated. The fast-flowering cultivar Italica Carolina was grown hydroponically up to the grain-filling stage. The plants were then treated with or without 500 μM of BSO for 5 d before panicles were excised for the As translocation assay. Five excised panicles from each of the –BSO or +BSO treatments were exposed to 10 μM of As(III) or 10 μM of DMA in the full-strength Kimura solution for 2 d. 10 μM RbCl and 10 μM SrCl_2_ were added to the solution as a tracer for phloem and xylem transport, respectively. 500 μM of BSO was also included in the +BSO treatment solution during the panicle experiment.

### Analysis of As, Rb, and Sr concentrations

Plant tissues were harvested and dried at 65 °C for 2 d. The samples were ground and digested in 5ml high purity HNO_3_/HClO_4_ (87/13, v/v). The concentrations of As, Rb, and Sr in the digests were determined by inductively coupled plasma mass spectrometry (ICP-MS, PerkinElmer NexION 300) operating in the helium gas mode to remove possible interference of ArCl on *m/z* 75 (As).

### Statistical analysis

Statistical significance was determined by one-way ANOVA using GenStat 16th edition (VSN International, Hemel Hempstead, UK).

## Results

### Arsenic distribution in node I of WT and the *lsi2-1* mutant

**Fig. 1.**
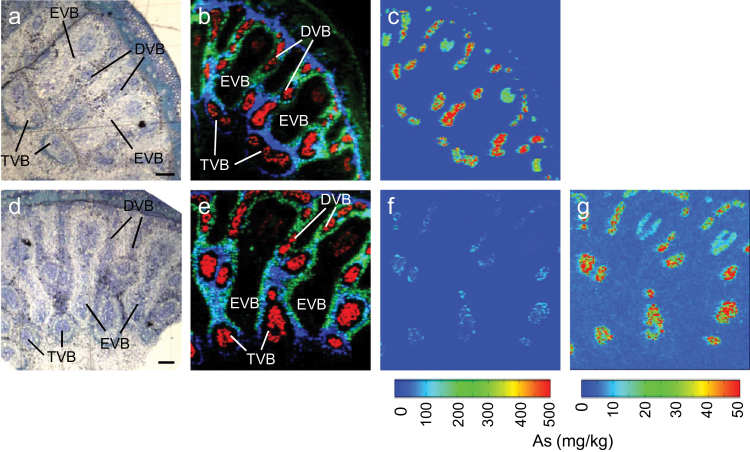
Synchrotron μ-XRF mapping images of As accumulation in node I of the WT (cv. T-65) and *lsi2-1*. Plants were grown in 2.0kg of Rothamsted soil supplied with 5 ppm of As(V) until the grain-filling stage. Node I was cut and prepared with high-pressure freezing, freeze-substitution, and embedding in resin. Samples for synchrotron μ-XRF were sectioned at 7 μm thickness and adjacent 1 μm sections were stained with Toluidine blue for optical images. (a, d) Optical images of the thin sections adjacent to the sections used for μ-XRF mapping for WT (a) and *lsi2-1* (d). (b, e) Colour merge showing the relative locations of As (red), Zn (green), and Fe (blue) in WT (b) and *lsi2-1* (e).(c, f, g) As concentrations across node I thin sections of WT (c) and *lsi2-1* (f, g). DVB, diffuse vascular bundle; EVB, enlarged vascular bundle; TVB, transit vascular bundle. Bars in (a) and (d)=100 μm.

Synchrotron μ-XRF was used to map the distribution pattern of As and other trace elements in the cross-sections of node I in *lsi2-1* and WT plants grown in soil to the grain-filling stage. The left panels in Fig. 1a and d show the optical images of the thin sections adjacent to the sections mapped by μ-XRF, showing different types of vascular bundles typical of rice node I (Yamaji and Ma, 2014). Figure 1b and e show composite images of As (red), zinc (Zn, green), and iron (Fe, blue), in the WT (Fig. 1b) and *lsi2-1* (Fig. 1e), respectively. The mapped regions covered two EVBs, a DVB, and a TVB. The relative scales used to display the As signal in Fig. 1b and e were very different because *lsi2-1* had a much lower signal intensity than the WT. For the Zn and Fe signals the relative scales were the same for the WT and *lsi2-1*. The three elements exhibited distinct distribution patterns, which were consistent with those reported recently for the rice node of a different cultivar (Moore *et al.*, 2014). Zn was strongly localized in the xylem parenchyma (XP) cells surrounding the EVBs and DVBs. Fe had a strong localization in the fundamental parenchyma (FP) cells close to the TVBs and the parenchyma cells outside of the EVBs. Arsenic was strongly localized to the phloem of EVBs, DVBs, and TVBs, with the signal being stronger in the latter two. The xylem regions (the dark regions surrounding the As signal within the vascular bundles) in all the vascular bundles were almost completely devoid of As signal. Although the As distribution pattern was similar between the WT and *lsi2-1* on the relative scale, on the same absolute scale As concentration was much lower in *lsi2-1* (Fig. 1f) than in the WT (Fig. 1c). The maximum As concentration detected among all scanned pixels was 2 177 and 409mg kg^–1^ for the WT and *lsi2-1*, respectively. Figure 1g displays As concentration in *lsi2-1* on a 10-fold lower range than that in Fig. 1c and f, showing a similar pattern of As distribution to that in the WT.

### Arsenic distribution in internode I of the WT and the *lsi2-1* mutant

**Fig. 2.**
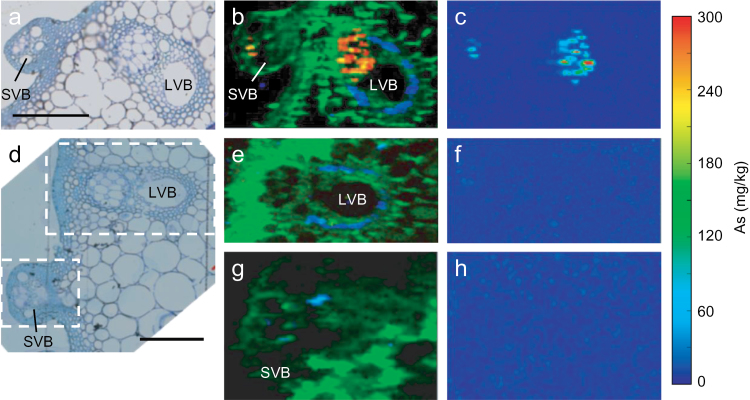
Synchrotron μ-XRF mapping images of As accumulation in WT (cv. T-65) and *lsi2-1* internode I (above node I). Internodes were sectioned at 7 μm thickness after high-pressure freezing, freeze-substitution, and embedding in resin. Adjacent 1 μM sections were stained with Toluidine blue for optical images. (a, d) Optical images of the thin sections adjacent to the sections used for μ-XRF mapping for WT (a) and *lsi2-1* (d). (b, e, g) Colour merge showing the relative locations of As (red), Zn (green), and Fe (blue) in WT (b) and *lsi2-1* (e, g). The squares in (d) show the mapped areas for *lsi2-1*. (c, f, h) As concentration across internode I sections of WT (c) and *lsi2-1* (f, h). LVB, large vascular bundle; SVB, small vascular bundle. Bars in (a) and (d)=50 μm.

Figure 2 shows the synchrotron μ-XRF images of the internode sections above node I. Figures 2a and d show the optical images of the thin sections adjacent to the mapped sections for the WT and *lsi2-1*, respectively. Large vascular bundle (LVB) and small vascular bundle (SVB) can be found in both sections. In the composite μ-XRF images, Zn was distributed relatively evenly in the entire mapped regions around the LVB and SVB of both the WT (Fig. 2b) and *lsi2-1* (Fig. 2e, g). By contrast, Fe was localized in a more specific region with a strong signal being observed in a single layer of the xylem parenchyma cells in the LVBs of the WT (Fig. 2c) and *lsi2-1* (Fig. 2e). Similar to that in node I, the xylem regions in all vascular bundles were devoid of the As signal. As accumulation was localized in the phloem regions in both the LVB and SVB of the WT internode (Fig. 2b, c). By contrast, the As signal was barely detectable in *lsi2-1* (Fig. 2e–h). The As concentration among all scanned pixels in the internode I specimen reached a maximum of 387mg kg^–1^ in the WT but only 85mg kg^–1^ in *lsi2-1*.

### *Lsi2* expression in different rice tissues

**Fig. 3.**
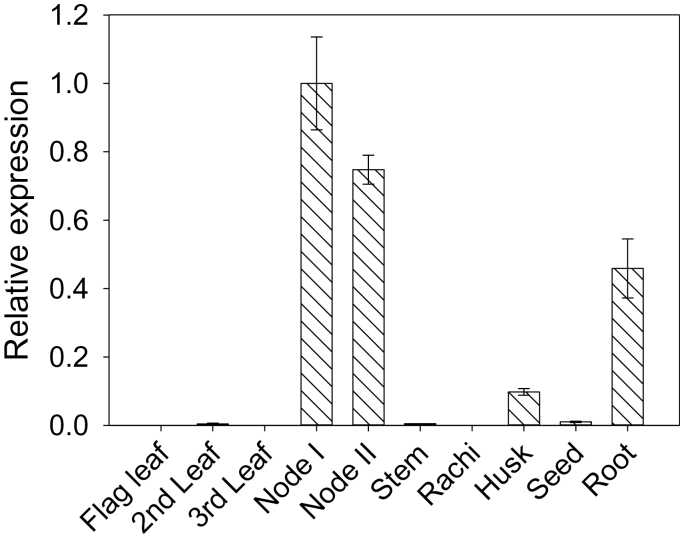
Relative expression of *Lsi2* in wild-type (cv. T-65) tissues at the grain-filling stage. Data are means ±SE.

*Lsi2* was previously shown to be highly expressed in rice roots (Ma *et al*., 2007, 2008). Here, the expression of *Lsi2* was quantified in different rice tissues at the grain-filling stage. Consistent with previous reports (Ma *et al*., 2007, 2008), *Lsi2* was found to be strongly expressed in roots (Fig. 3). There was little expression of *Lsi2* in leaf, internode, rachis, and seed materials. However, there was strong expression of *Lsi2* in both nodes I and II, with the transcript abundance being even higher than that in the roots. *Lsi2* expression was also found in the husk, but at only at one-fifth of the level in the roots.

### The role of *Lsi2* in As(III) distribution in panicle tissues

**Fig. 4.**
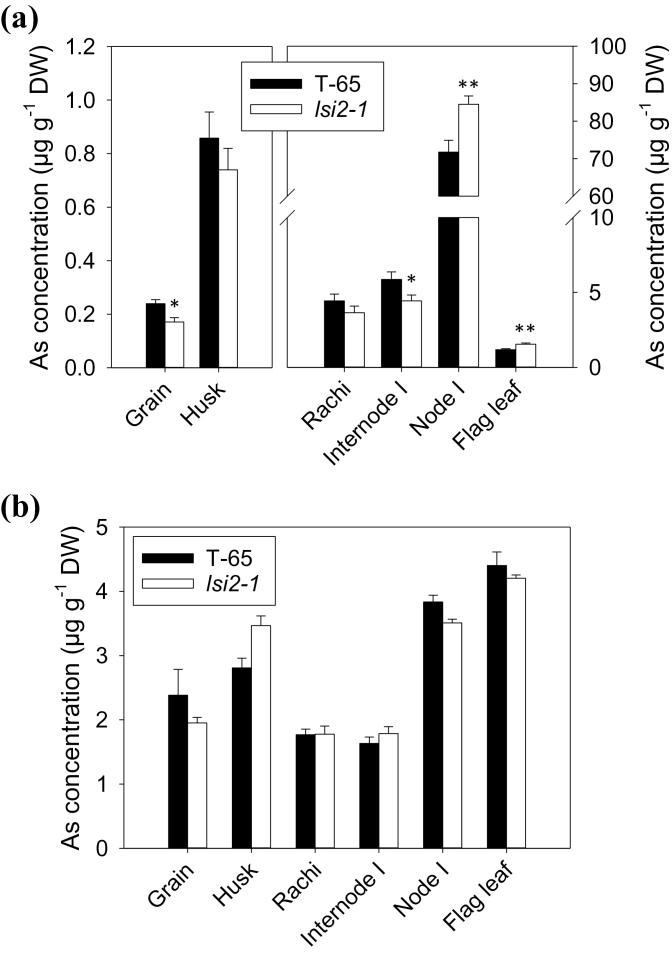
Arsenic concentrations in different panicle tissues of WT (cv. T-65) and *lsi2-1* mutants exposed to 10 μM As(III), RbCl and SrCl_2_ (a) or to 5 μM of DMA, 10 μM RbCl and SrCl_2_ (b). Data are means ±SE. *^,^**, Significant difference between WT and mutant at *P* <0.05 and *P* <0.01, respectively.

Based on the observation of a high *Lsi2* expression in the node, its role in As distribution was investigated using excised panicles (including the flag leaf and node I). To achieve this, *lsi2-1* and WT panicles were cut from the internode below node I from plants pre-grown in hydroponic culture free of As. The excised panicles were then fed through the cut end with As(III) or DMA in the nutrient solution. DMA was included for a comparison as a previous study showed that it is not permeable through Lsi2 (Li *et al.*, 2009a). In the As(III) treatment, the loss of Lsi2 function in the *lsi2-1* mutant resulted in significantly (*P* <0.01) increased As accumulation in node I and the flag leaf, but significantly decreased As accumulation in internode I and the grain (*P* <0.05) compared with the WT (Fig. 4a). There was no significant difference between *lsi2-1* and WT in As accumulation in the rachis and husk. The concentration of As accumulated in the node was much greater than that in other tissues. For example, the ratio of As concentration in node I to that in the flag leaf and grain was 60.1 and 306.4, respectively, in the WT. The *lsi2-1* mutant had a similar ratio of node I to flag leaf As concentration (55.6), but a significantly (*P* <0.01) higher ratio of node I to grain As concentration (522.4) than the WT. In contrast to the As(III) treatment, there was no significant difference in As distribution between WT and *lsi2-1* when DMA was fed to the excised panicles (Fig. 4b). In addition, DMA was more evenly distributed to different tissues of the rice plants, with a variation in the tissue As concentration of approximately 2-fold only and the flag leaf having a slightly higher concentration than node I. For both WT and *lsi2-1*, the grain As concentration was more than 10 times higher than that in the As(III) treatment. Rubidium (Rb) and strontium (Sr) were added to the nutrient solution as markers for phloem and xylem transport, respectively (Kuppelwieser and Feller, 1991). There was no significant difference between *lsi2-1* and the WT in the concentrations of either element in all tissues (see Supplementary Fig. S1 at *JXB* online), suggesting that Lsi2 mutation does not affect either xylem or phloem transport.

### The effect of BSO on As distribution in excised panicles

**Fig. 5.**
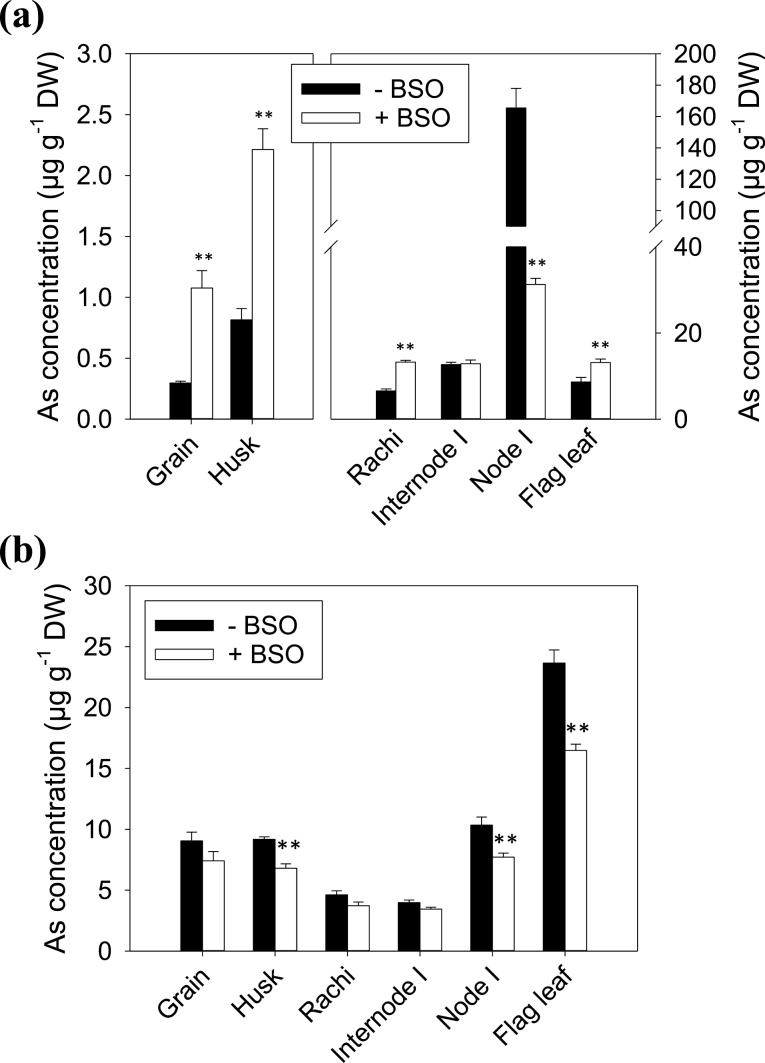
Arsenic concentrations in different panicle tissues of cv. Italica Carolina exposed to 10 μM As(III), RbCl and SrCl_2_ (a) or to 5 μM DMA, 10 μM RbCl and SrCl_2_ (b) with or without l-buthionine-sulphoximine (BSO). Data are means ±SE. *^,^**, Significant difference between +BSO and –BSO treatments at *P* <0.05 and *P* <0.01, respectively.

The strong accumulation of As in the phloem of different vascular tissues in the node (Fig. 1) and the co-localization of As and S in these tissues (Moore *et al.*, 2014) suggest that thiol compounds such as PCs and GSH may play an important role in As storage and distribution. To manipulate the biosynthesis of thiol compounds, BSO was fed to rice plants (cv. Italica Carolina) at the grain-filling stage and excised panicles were used in an As distribution experiment. In the As(III) treatment, the +BSO treatment markedly decreased the As concentration in node I by 81% (*P* <0.001) compared with the –BSO control (Fig. 5a). By contrast, +BSO significantly (*P* <0.001) increased the concentration of As in the flag leaf (by 52.7%), rachis (by 101.2%), husk (by 171.1%), and the grain (by 263.5%). The ratio of grain to node As concentration was 0.0018 and 0.0341 in the –BSO and +BSO treatments, respectively (*P* <0.001), whilst the ratio of flag leaf to node As concentration was 0.052 and 0.425, respectively (*P* <0.001). In the DMA treatment, +BSO significantly decreased As concentrations in the flag leaf (by 30.4%), node I (by 25.1%), and husk (by 25.9%) (Fig. 5b), but the effect was relatively small and distinctively different from that observed in the As(III) treatment. There was no significant difference in the ratio of grain to node As concentration in the –BSO (0.875) and +BSO (0.963) treatments. Similarly, the ratio of flag leaf to node As concentration was also not statistically different in –BSO (2.33) and +BSO (2.16) treatments. The concentrations of Rb and Sr in some of the tissues were also significantly decreased by +BSO (see Supplementary Fig. S2 at *JXB* online), suggesting that the BSO treatment might affect the general metabolism, resulting in slightly decreased uptake of Rb, Sr, and DMA.

## Discussion

Recent studies have identified rice nodes as the critical hub in controlling the distribution of mineral elements (Yamaji and Ma, 2014). In the top node, EVBs and DVBs are connected to the flag leaf and the panicle, respectively, and the transfer of minerals between these two types of vascular bundle determines the relative distribution of minerals between the flag leaf and the grain. Nodes have a markedly larger concentration of As than the other tissues of rice shoots (Moore *et al.*, 2014). This was confirmed in the present study using a short-term exposure of excised panicles to As(III), showing that the top node accumulated As to a concentration 1–2 orders higher than in the other tissues (Fig. 4). The reason for this massive As accumulation is likely to be due to the presence of many vascular bundles in the node, where As accumulates strongly in the phloem as revealed by synchrotron μ-XRF mapping (Fig. 1). Further investigation using NanoSIMS revealed that As is stored inside the vacuoles of the companion cells in the phloem with a strong co-localization of As and S (Moore *et al.*, 2014). Such co-localization is indicative of vacuolar As sequestration in the form of As–thiol complexes. Recently, Song *et al.* (2014) identified an As(III)–phytochelatin transporter in rice, OsABCC1, that is localized to the tonoplast of the phloem companion cells in the rice node. When this transporter was knocked out, less As accumulated in the node and more As was distributed to the grain. In the present study, it was found that the inhibition of thiol synthesis by BSO markedly decreased As accumulation in the node, but increased As distribution to the flag leaf and the grain (Fig. 5). Taken together, the results suggest that rice nodes serve as an important filter of As(III), restricting its movement to the grain and flag leaf. This is achieved by the synthesis of thio compounds, such as phytochelatins, and transport of As(III)–thiol complexes into the vacuoles of the companion cells in the phloem of rice nodes. The phloem companion cells in the internode may also play a similar role (Fig. 2), but the effect is small compared with the node because they represent a much smaller proportion of all the cells in the internode than in the node.

Ma *et al.* (2008) have previously shown that the silicic acid efflux transporter Lsi2 plays an important role in the translocation of As(III) from rice roots to the shoots. In both hydroponically and field-grown plants, the *lsi2-1* mutant accumulated much lower As concentrations in the shoots than WT plants (Ma *et al.*, 2008). In the present study, μ-XRF showed markedly lower As accumulation in the phloem of various types of vascular bundles in both the top node and internode of the *lsi2-1* mutant than in WT plants grown in soil pots (Figs 1, 2). This difference can be largely attributed to the function of Lsi2 in transporting As(III) out of the exodermal and endodermal cells of the roots towards the stele for xylem loading. In addition, *Lsi2* was found to be highly expressed in the node as well as in the roots (Fig. 3), suggesting a possible role of Lsi2 in As(III) distribution to the panicle. In the nodes of barley, Lsi2 was found to be localized to the parenchyma cell bridge bordering EVB and DVB (Yamaji *et al.*, 2012). To separate the possible role of Lsi2 in the node from that in the roots, As(III) was fed to excised panicles cut below the top node in a short-term exposure experiment. Compared with the WT, the *lsi2-1* mutant accumulated more As(III) in the node and the flag leaf, but less As(III) in the grain and internode I above the top node (Fig. 4). This altered distribution pattern is consistent with a role of Lsi2 in transferring As(III) from EVBs to DVBs, thus affecting the relative distribution of As(III) to the grain and flag leaf.

In addition to inorganic As (mainly As(III)), the rice grain also contains methylated As species, particularly DMA (Meharg *et al.*, 2009; Zhao *et al.*, 2013). The source of DMA is probably soil microbes because rice plants lack the ability to methylate As (Lomax *et al.*, 2012). Compared with As(III), DMA has a much greater mobility within rice plants (Li *et al.*, 2009a; Carey *et al.*, 2011; Lomax *et al.*, 2012). This is confirmed in the short-term exposure experiments with excised panicles (Figs 4, 5). The concentrations of As in the grain and flag leaf were much greater in the DMA treatment than in the As(III) treatment, whereas the node did not preferentially accumulate DMA as in the As(III) treatment. Lsi2 mutation did not affect the distribution of DMA, unlike that of As(III) (Fig. 4). This is expected because Lsi2 was found not to be permeable to DMA (Li *et al.*, 2009a). Furthermore, BSO had no significant effect on the relative distribution of DMA between the node, flag leaf, and grain (Fig. 5), also unlike that for As(III). This suggests that DMA is not complexed by thiol compounds such as phytochelatins. Using parallel HPLC-ICP-MS/ES-MS detection, Raab *et al.* (2007) identified a number of As(III)–PC and MMA–PC complexes in sunflower plants, but no DMA–PC complexes were found. The lack of DMA–thiol complex formation may explain the high mobility of DMA in plants because DMA is not sequestered in the vacuoles of the phloem companion cells as is As(III).

In conclusion, the present study has shown that rice nodes serve as a filter of inorganic As, restricting its movement to the grain, with As preferentially accumulating in the phloem within different types of vascular bundles. The silicic acid/arsenite transporter Lsi2 plays a role in As(III) distribution in the node. Inhibiting the synthesis of thiol compounds suppresses the filtering role of the node for As(III), but not DMA. Enhancing the synthesis of thiol compounds or the transport of As(III)–thiol complexes into the vacuoles in the phloem companion cells may be a promising strategy to restrict As(III) accumulation in the rice grain.

## Supplementary data

Supplementary data can be found at *JXB* online.

Supplementary Fig. S1. The concentrations of Rb (a) and Sr (b) in panicle tissues of WT (T-65) and the *lsi2-1* mutant exposed to 10 μM of As(III), RbCl, and SrCl_2_.

Supplementary Fig. S2. The concentrations of Rb and Sr concentrations in panicle tissues of rice cv. Italica Carolina exposed to 10 μM of As(III), RbCl, and SrCl2 (a) or 5 μM of DMA, 10 μM RbCl and SrCl_2_ (b) with or without l-buthionine-sulphoximine (BSO).

Supplementary Data
