# Supplementary material for: The role of nodes in arsenic storage and distribution in rice
Source: J Exp Bot. 2015 Apr 28;66(13):3717–24. doi: 10.1093/jxb/erv164 (PMC4473974; doi:10.1093/jxb/erv164)
Supplement: Supplementary Data [file supp_66_13_3717__index.html]

The role of nodes in arsenic storage and distribution in rice — The role of nodes in arsenic storage and distribution in rice — Supplementary Data 

# The role of nodes in arsenic storage and distribution in rice

## Supplementary Data

Data files

**Files in this Data Supplement:**

- Supplementary Data - Supplementary Data
