# Supplementary material for: The role of nodes in arsenic storage and distribution in rice
Source: J Exp Bot. 2015 Apr 28;66(13):3717–24. doi: 10.1093/jxb/erv164 (PMC4473974; doi:10.1093/jxb/erv164)
Supplement: Supplementary Data [file supp_erv164_jexbot143735_file001.pdf]

The role of nodes in arsenic storage and distribution in rice

Yi Chen, Katie L. Moore, Anthony J. Miller, Steve P. McGrath,
Jian Feng Ma and Fang-Jie Zhao

Supplementary data

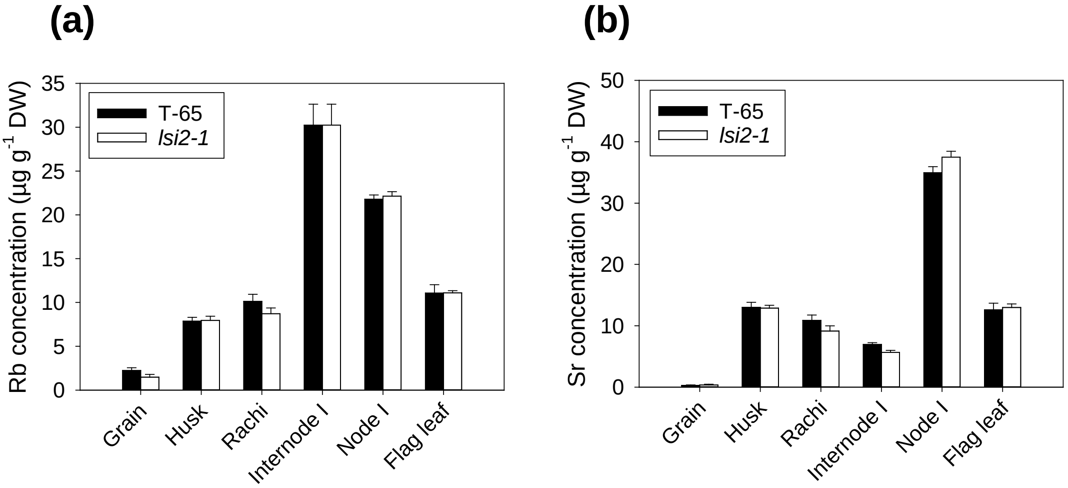

Figure S1. The concentrations of Rb (a) and Sr (b) in panicle tissues of WT (T-65) and *lsi2-1* mutant exposed to 10 µM of As(III), RbCl and $SrCl_2$. Data are means ± S.E.

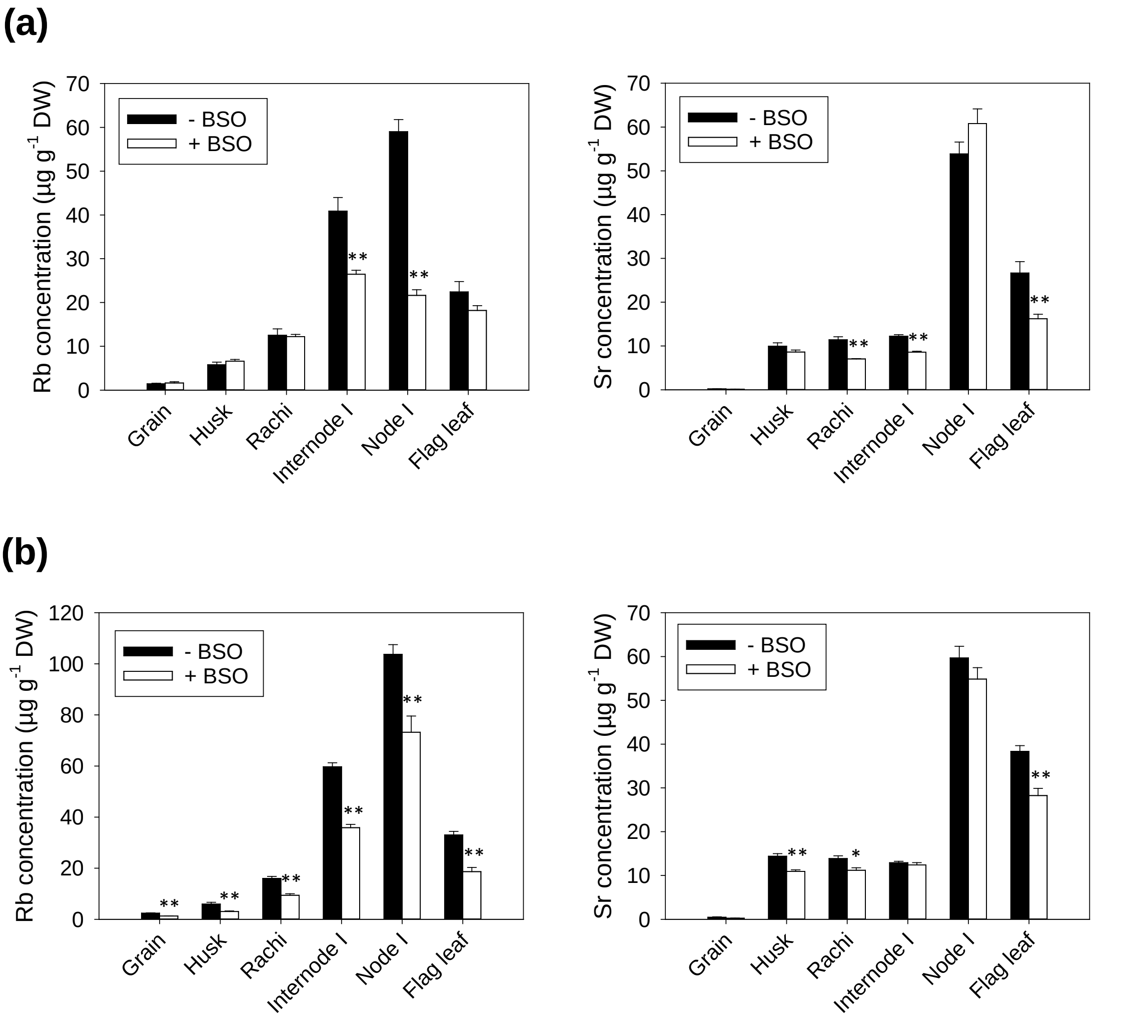

Figure S2. The concentrations of Rb and Sr concentrations in panicle tissues of rice cv. Italica Carolina exposed to 10 µM As(III), RbCl and SrCl2 (a) or 5 µM DMA, 10 µM RbCl and SrCl2 (b) with or without L-buthionine-sulphoximine (BSO). Data are means ± S.E. * and **, significant difference between –BSO and +BSO treatments at $P<0.05$ and $P<0.01$, respectively.
